# Supplementary material for: Causal interplay between lactose intolerance and gut microbiota: a combined bidirectional Mendelian randomization and in vivo validation study
Source: Front Nutr. 2026 Jun 1;13:1803337. doi: 10.3389/fnut.2026.1803337 (PMC13265576; doi:10.3389/fnut.2026.1803337)
Supplement: Supplementary file 1 [file Data_Sheet_1.zip › supplementary materials/Forward/forest plot/ebi-a-GCST90027836.finngen_R12_E4_LACTONAS.pdf]

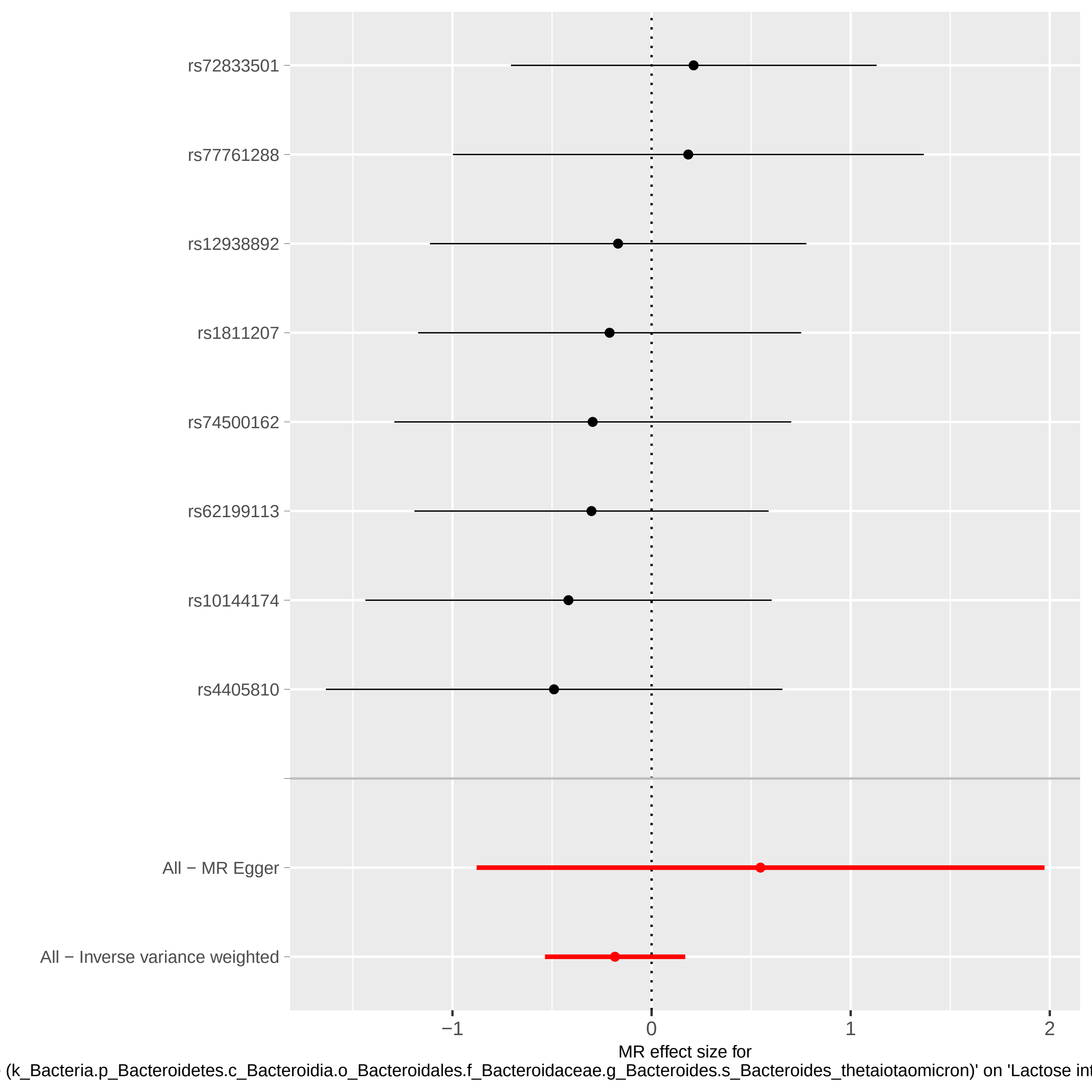

rs72833501
rs77761288
rs12938892
rs1811207
rs74500162
rs62199113
rs10144174
rs4405810
All − MR Egger
All − Inverse variance weighted
−1
0
1
2
MR effect size for
(k_Bacteria.p_Bacteroidetes.c_Bacteroidia.o_Bacteroidales.f_Bacteroidaceae.g_Bacteroides.s_Bacteroides_thetaiotaomicron)' on 'Lactose int
